# Maternal insecure attachment representation as a long-term risk factor for disrupted mother–child-interaction and child mental health

**DOI:** 10.1186/s40359-024-01874-8

**Published:** 2024-07-09

**Authors:** Katharina Rost, Ute Ziegenhain, Jörg M. Fegert, Anna Buchheim, Franziska Köhler-Dauner

**Affiliations:** 1 Department of Child and Adolescent Psychiatry/Psychotherapy, University Hospital of Ulm, Ulm, Germany; 2 German Center for Mental Health (DZPG), partner site Ulm, Ulm, Germany; 3 Institute of Psychology, University of Innsbruck, Innsbruck, Austria

**Keywords:** CM experience, Maternal attachment, Mother–child-interaction, Maternal helplessness, Child mental health

## Abstract

Maternal childhood maltreatment (CM) represents an important factor in the transmission of trauma that may lead to impaired child mental health. Apart from childhood maltreatment insecure attachment has been identified as a risk factor for insensitive caregiving behavior, which may affect child's mental health. The aim of this study is to identify the working mechanisms in the relationship between maternal CM and child mental health, considering maternal attachment representation, mother–child-interaction und maternal helplessness and fear. *N* = 103 mother–child-dyads from a longitudinal cohort study were examined at four different measuring points. Data was assessed using self and external report questionnaires as well as the *AMBIANCE* scales during the Strange Situation Procedure and the *Adult Attachment Projective Picture System* (*AAP*). Maternal CM experience did not predict an insecure attachment representation (*OR* = 2.46 [0.98, 6.53], *p* = .060). Maternal insecure attachment was associated with higher *AMBIANCE* scores (*F*(8, 94) = 11.46, *p* < .001), which indicates more disrupted communication between mother and child. *AMBIANCE* scores in turn predicted higher self-perceived helplessness (*F*(9, 93) = 8.62, *p* < .001) and fear (*F*(9, 93) = 7.40, *p* < .001) in mothers. Helplessness and fear both were associated with higher *SDQ*-scores, indicating more mental health problems in children (*F*(10, 92) = 3.98, *p* < .001; *F*(10, 92) = 3.87, *p* < .001). The results of this study highlight how even insecure attachment in a low-risk sample has a long-term impact on parenting behavior and child mental health, therefore underlining the need of early intervention programs in affected and at-risk families.

## Introduction

Childhood maltreatment (CM) is associated with many negative effects, which can manifest until adult life. For example, associations of CM with mental disorders in adulthood have been reported [1, 2]. In particular, the negative effects of CM become clear when affected individuals become parents themselves. Already shortly after birth, mothers with CM experiences suffer more often from postpartum depression [3]. Additionally, mothers with CM experiences are more likely to abuse their own children themselves, which is referred to in the literature as the “cycle of maltreatment” [4, 5]. It is assumed that children with traumatized mothers grow up in a more stressful environment and may not always have their needs addressed adequately and thus passing on across generations [6]. CM influences various qualities of parenting such as sensitivity, satisfaction, availability, time spent with the child, coping with stressful situations and perceived ineffectiveness [5–8]. Furthermore, abused mothers spend less time, are less satisfied with their children, and report higher self-perceived inability to parent [7]. Similarly, attachment functions play an important role in the transgenerational transmission of trauma, as traumatized parents have a harder time recognizing their children's needs [9, 10]. Additionally, many findings confirm that CM experiences affect attachment representation within adulthood (e.g. [11]). A study by Widom and colleagues [12] shows that individuals with CM experiences are more likely to exhibit a fearful or avoidant attachment type compared to individuals without abusive experiences. Moreover, physical abuse and frequent experiences of abuse in general, are associated with insecure attachment types and more pronounced insecure relationship patterns [13, 14].

Insecure attachment representation on the other hand also predicts lower-quality parenting behaviors and may be considered a risk factor for disruptive parenting behavior. Previous studies were able to identify associations of insecure attachment representation with less sensitivity, more intrusive parenting behavior and high levels of disorganization, which impair caregiving behavior [15, 16]. Additionally, insecure attachment representation is associated with increased stress, which can also have a negative impact on parenting behavior. On the contrary, mothers with secure attachment representation are in general more capable to cope with becoming a mother [17]. Therefore, in summary, insecure attachment representation can represent a risk factor for many components of sensitive parenting behavior.

Another factor that may play a role in this context is caregiving helplessness and fright, which can be assessed by the Caregiving Helplessness Questionnaire (*CHQ*; [18]). Previous studies were able to show that caregiving helplessness and fright could predict the child's attachment [19] and parental perception of infant socioemotional problems [20] at approximately 12 months old as well as child mental health at the time of school entry [21]. Moreover, caregiving helplessness was shown to mediate the effect of maternal trauma symptoms on parental harsh discipline towards children (e.g. shaking the child, hitting the child on the bottom; [22]). Likewise, the role of self-efficacy, which could be related to helplessness in parenting behavior has been examined in current literature. Previous studies examined that maternal CM experiences, parental abuse in childhood, maternal attachment insecurity and parental avoidant attachment representation were associated with low self-efficacy in raising children [23, 24]. Concerning the role of parental fear, studies showed that both maternal and paternal parenting behavior seem to play a role in the development of child anxiety [25]. Additionally, trait anxiety in mothers predicted parental stress after birth [26]. Stress in parents may in turn affect child's mental health, as maternal stress has been identified as a predictor for emotional [27], behavioral [28] and externalizing behavior problems in children [29].

**Fig. 1:**
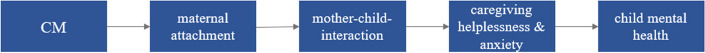
Exploratory path model

The purpose of this study is to further examine the pathways between maternal CM experiences and their child’s mental health. Previous studies were able to show several connections between CM experiences, attachment style, parenting behavior, parental anxiety and fear and child’s mental health. However, the exact relationships between the variables over time have not yet been sufficiently investigated. Additionally, in this study, the construct of helplessness and fright in raising children should be considered. Based on existing findings, it can be assumed that helplessness and anxiety on the part of the mother have an influence on children mental health, which is why these concepts are examined in more detail. Based on the existing literature, a pathway model which will be exploratively analyzed in this study (Fig. 1). Specifically, we will examine if maternal CM experiences have an impact on maternal attachment representations. Moreover, the effect of maternal attachment representation on mother-infant interactions will be examined. Furthermore, the influence of mother–child interaction on caregiving helplessness and anxiety and their impact on children’s mental health will be investigated.

## Methods

### Study design and recruitment of participants

Within the project Trans-Gen, mother–child dyads were examined longitudinally starting from birth. The recruitment of mother–child dyads (*N* = 533) took place in the women´s hospital of the University hospital of Ulm shortly after parturition and started in October 2013 within 1 to 6 days after parturition (time point t_0_). Mothers provided written informed consent before participating in the study. After, they took part in an initial screening interview. CM load was assessed using the *Childhood Trauma Questionnaire* (*CTQ*; [30]). All mother–child dyads were invited to the university at three sequential time points: 3 months (t_1_), 12 months (t_2_), and 24 to 36 months after birth (t_3_). With *N* = 279 mother–child dyads, maternal attachment was assessed using the *Adult Attachment Projective Picture System* (*AAP*; [31]) at t_1_. The following data regarding the *Atypical Maternal Behavior Instrument for Assessment and Classification (AMBIANCE)* scale [32] were assessed at t_2_ (*N* = 246). For data collection, all mother–child dyads were invited to the Department of Child and Adolescent Psychiatry/Psychotherapy at the Ulm University Hospital. At the next measurement time point (t_4_) *N* = 116 mothers were visited in their homes, where caregiving helplessness and child mental health were assessed via questionnaires using the *Caregiving Helplessness Questionnaire* (*CHQ*; [18]) and the *Strengths and Difficulties Questionnaire* (*SDQ*; [33]).

### Participants

In total, *N* = 103 mother–child dyads participated at time point t_0_, t_1_, t_2_ and t_4_. The average age of participating mothers was *M* = 38.43 years (*SD* = 4.22), ranging from 30 to 47 years. Educational years were measured as an ordinal variable (1 =  ≤ *9 years of education*, 2 = *10 years of education* and 3 =  ≥ *12 years of education*), 73% of all mothers reported 12 years of education or more, whereas 20% reported 10 years of education, and 7% reported 9 years of education or less. The average age of all children was *M* = 5.30 years (*SD* = 0.52) with their ages ranging from 4 to 7 years. Almost half of all children (49%) were females (Table 1).

**Table 1:** Descriptive statistics of all variables (*N* = 103)

	***M***	***SD***	***Range***
age mother at t_4_	38.43	4.22	30–47
age child at t_4_	5.30	0.52	4–7
CHQ			
helplessness	12.34	4.32	7–29
fear	9.61	3.48	6–19
role reversal	18.05	3.22	10–25
AMBIANCE global scale	4.29	1.27	1–7
SDQ			
emotional problems	1.52	1.50	0–5
conduct problems	2.13	1.52	0–7
hyperactivity scale	2.34	2.09	0–9
peer problems scale	1.07	1.55	0–7
prosocial scale	7.50	1.87	1–10
PSS-14	22.33	9.57	3–44
	***N***	***%***	
CM +	49	48	
AAP			
secure	35	34	
insecure	68	66	
sex (male)	53	51	
years of education			
≤ 9 years	7	7	
10 years	21	20	
≥ 12 years	75	73	

## Measures

### Childhood maltreatment (CM)

Maternal childhood maltreatment experiences were assessed at t_0_ using the German short version of the Childhood Trauma Questionnaire (*CTQ*; [30]). The *CTQ* screening assesses the child maltreatment through a retrospective self-report. It contains five subscales each assessed by 5 items on a 5-point Likert scale, screening emotional, physical and sexual abuse as well as physical and emotional neglect. Additionally, three items assess whether participants tend to trivialize problematic experiences. Internal consistency in a German sample ranges between 0.62 and 0.96 [34]. Severity scores for each subscale as well as a total score including all five subscales can be calculated, ranging from “none maltreatment experiences” (CM-) over “minimal” to “extreme” maltreatment load (CM +) Mothers with a total score ≥ 6 were declared as CM + .

### Adult Attachment Projective Picture System (AAP)

Maternal attachment representation was assessed at t_1_ using the Adult Attachment Projective Picture System (*AAP*; [31]). The AAP is a standardized, objective, reliable and valid attachment interview using eight-line drawings. After the drawings were presented, a standardized set of questions was asked encouraging the participant to tell a story about each picture. The first neutral warm-up picture, is followed by seven drawings depicting attachment-related scenes (e.g., separation, illness, loss, and potential maltreatment). These seven stimuli are designed to activate the participant’s attachment system. The participant’s audio-recorded responses are evaluated considering content, discourse and defensive processes along the manual [30]. In the *AAP*, the attachment representation is expressed by assigning to one of the four attachment classifications: "Secure attachment", "Insecure-distant attachment", "Insecure-entangled attachment" and "Unprocessed trauma" [31]. However, the *N* in the individual attachment classifications was too low to evaluate them individually, therefore the study only distinguishes between "secure attachment" and a combination of the other classifications summarized in "insecure attachment". Therefore, only the two superordinate classes are referred to in the analysis of the data of this study. All interviews were conducted by trained psychologists. *AAP* classifications were coded by two independent certified judges. The reliability and validity of the *AAP* could be confirmed in the extensive psychometric validation study by George and West (interjudge-reliability *r* = 0.70-0.89, retest-reliability *r* = 0.70) [31] with an agreement of 90% between *AAP* and *Adult Attachment Interview* (*AAI*; [35]) regarding the four attachment groups. The convergent and discriminant validity of the *AAP* was also confirmed by a study by Beliveau and Moss (2005) [36].

### AMBIANCE scales

All Strange Situation Procedure sessions were videotaped at t_2_ to analyse the quality of maternal interactive behaviour between the mother and her infant using the *AMBIANCE* [37]. The *AMBIANCE* instrument is based on Main and Hesse's theory, which explains the frightened, frightening, and dissociated parental behaviour [38]. Therefore, they considered profound disruptions in mother–child interactions and emotional as well as physical withdrawal behaviours such as anomalous parental behaviour of mothers during the interactions with their children. The *AMBIANCE* is a coding system that assesses disrupted maternal behaviours on five dimensions on a 7-point scale: 1) affective communication errors, 2) role/boundary confusion, 3) disorganized/ disoriented behaviours, 4) negative/intrusive behaviour, and 5) withdrawal. For a final assessment, the overall score of the general level of disruption is determined. This score is based on the displayed intensity and frequency of disrupted behaviours during the recorded mother–child interaction, whereby a level of disrupted communication of up to 4 is considered “not-disrupted” and a level of 5–7 is considered “disrupted”. In this study, only the final score of the *AMBIANCE* is included in the analyses. A single coder, who was trained by and reliable according to the original developers of the *AMBIANCE*, scored all play sessions blinded to the data sets of the mother-infant dyads [36]. In a previous study interrater-reliability between two coders ranged from *r*_i_ = 0.73–0.84 [33].

#### CHQ

The Caregiving Helplessness Questionnaire (*CHQ*) was used during t4 in-home-visit as a self-report screening tool for disorganized caregiving. George and Solomon [18] developed the *CHQ* to provide an efficient method of assessment. The questionnaire aims to measure the dimensions of “caregiving helplessness”, “fear” in the relationship between parent and child and the parent–child “role reversal”. The *CHQ* includes 26 items divided into three subscales: seven items in the “caregiving helplessness” subscale (e.g. “*When I am with my child, I often feel out of control*”), six items in the “fear” subscale (e.g. “*Sometimes my child acts as if he/she is afraid of me*” and “*I am frightened of my child*”) and another six items in the “role reversal” subscale (e.g. “*My child is good at tending to and caring for others*”). Seven other items are fillers. The items are rated on a 1–7 scale (not characteristic at all – very characteristic). Higher scores represent a stronger manifestation of the given construct. George and Solomon [18] reported a good factor structure and adequate internal reliability, with alpha coefficients of α = 0.85, α = 0.66 and α = 0.64 as well as good convergent validity. Internal consistencies in our sample were measured at *α*_helplessness_ = 0.70, *α*_fear_ = 0.57 and *α*_role reversal_ = 0.45.

### Strengths and Difficulties Questionnaire (SDQ)

The German version of the Strengths and Difficulties Questionnaire (*SDQ*; [33]) was used at t_4_ to survey the children’s mental health and was completed by their parents. The questionnaire contains five scales, each scale consists of five items, which are rated on a 3-point Likert scale (0 = not applicable, 1 = partially applicable, 2 = clearly applicable). The five scales are “emotional problems” (e.g. “*Many worries or often seems worried*” and “*Many fears, easily scared*”), “conduct problems” (e.g. “*Often loses temper*” and “*Often lies or cheats*”), “hyperactivity scale” (e.g. “*Restless, overactive, cannot stay still for long*” and “*Easily distracted, concentration wanders*”) “peer problem scale” (“*Rather solitary, prefers to play alone*” and “*Gets along better with adults than with other children*”) and “prosocial scale” (e.g. “*Considerate of other people's feelings*” and “*Often offers to help others (parents, teachers, other children*”). For each scale, the sum score of all 5 items was calculated. Higher values indicate more severe problems. Husky and colleagues [39] report satisfactory internal consistencies (α _Emotional Problems_ = 0.74, α _Conduct problems_ = 0.74, α _Hyperactivity scale_ = 0.82, α _Peer problem scale_ = 0.67 α _Prosocial scale_ = 0.71) in a German sample. Internal consistencies in our sample were measured at *α*_Emotional Problems_ = 0.61, *α*_Conduct problems_ = 0.57, *α*_Hyperactivity scale_ = 0.77, *α*_Peer problem scale_ = 0.69 and *α*_Prosocial scale_ = 0.69.

### Perceived daily stress (PSS-14)

The mothers' daily stress as perceived by them was assessed via the Perceived Stress Scale 14 [40] at t_1_, t_2_ and t_4._ The *PSS-14* as the original version measures perceived stress in the previous four weeks. It consists of a 5-point scale from 0 to 4 within a total of 14 items of which 7 are positive and 7 are negative. After the positive items are reversed, a sum score can be calculated from all 14 items. The sum score can range from 0 to 54. In general, high scores are an indicator of high degrees of perceived stress, however, there are no cut-off values as the *PSS* is not a diagnostic tool. Previous studies have found high internal consistency between *α* = 0.82 to *α* = 0.86, as well as evidence for the convergent, concurrent and criterium validity of the scale [40–42]. The internal consistency in our sample is α = 0.92.

### Statistical analyses

Data were analyzed using R version 4.1.3 [43] and *p*-values ≤ 0.05 were considered significant. Some mothers who participated at t_4_ did not have complete data from the previous survey time points (*n* = 15) and therefore had to be excluded, resulting in *N* = 103 participants. The path model was calculated using multiple regressions and one binary logistic regression. Age of the mother, the age of the child, the mother’s education years (≤ 9 years, 10 years, ≥ 12 years) and the mothers stress level at the respective time of measurement (*PSS-14* sum score) were included as control variables in every model. A total of 23 regression models were calculated. For all multiple regression models, assumptions of linear regression were checked visually and analytically. Due to heteroscedasticity in some models, heteroscedasticity consistent (HC) standard errors were calculated in affected models via the "lmtest" package in R. Depending on the normal distribution of the residuals and the presence of outliers, HC3 or HC4 estimators were calculated [44]. A a-priori power analysis was conducted using the program G*Power (Version 3.1.9.4). Medium effects were expected. Depending on the number of predictors (*n* = 6–9), the required sample size for a power of *p* = 0.9 was between *N* = 123 and *N* = 141.

## Results

### Descriptive analyses

Descriptive statistics of demographic variables and all variables included in the path analysis are provided in Table 1.

### Path analysis

**Fig. 2:**
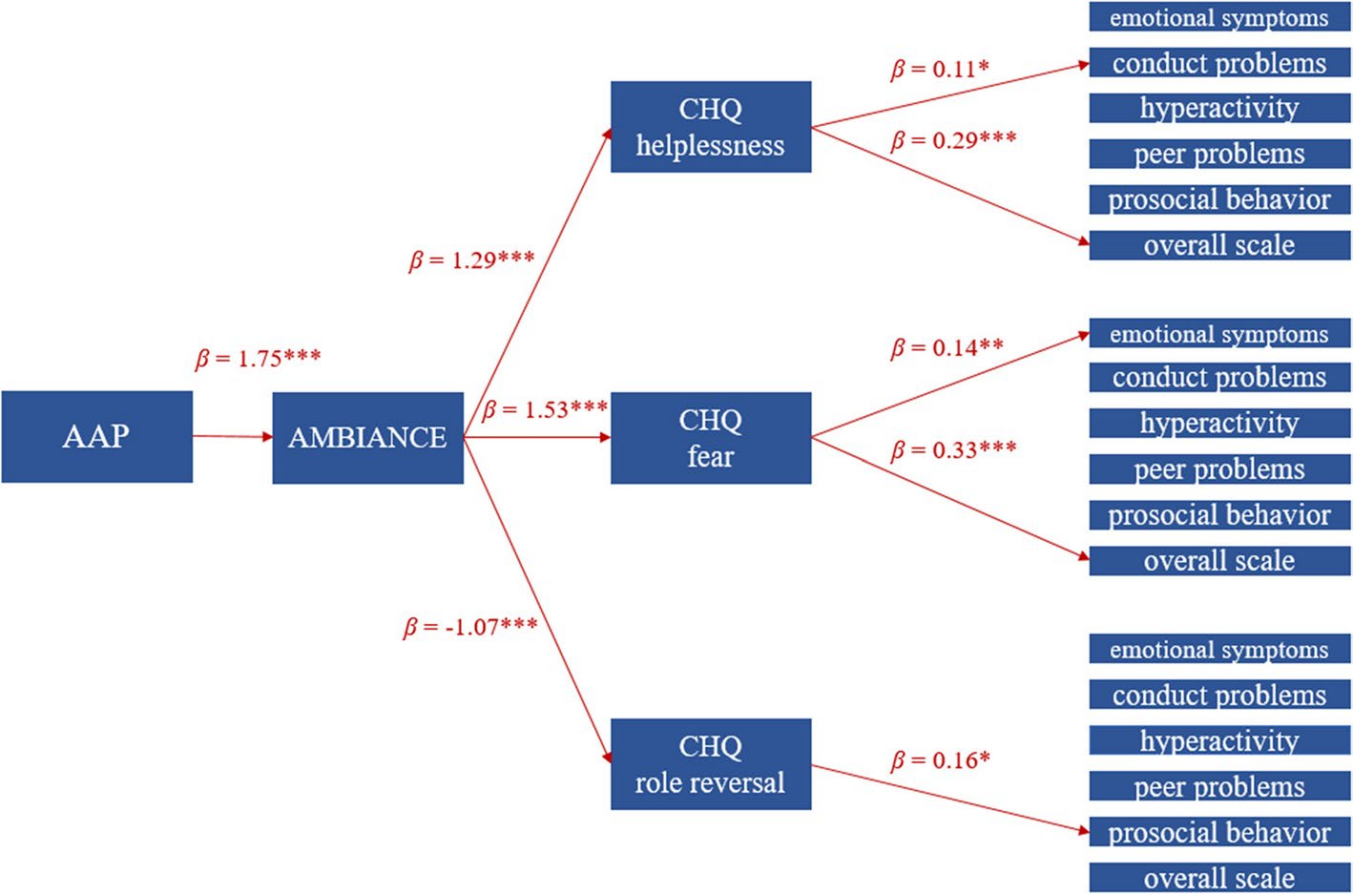
Overview of all confirmed paths of the path analysis *Note.* AAP = Adult Attachment Projective Picture System, AMBIANCE = Atypical Maternal Behavior Instrument for Assessment and Classification System, CHQ = Caregiving Helplessness Questionnaire

The results of all models of the path analysis are shown in Tables 2, 3, 4, 5, 6 and 7. An overview of all confirmed paths of the path analysis is illustrated in Fig. 2. In the first path analysis model, CM experience was not a significant predictor of maternal attachment representation. Mothers with CM experiences did not have an increased likelihood of insecure attachment representation (*OR* = 2.46 [0.98, 6.53], *SE* = 0.48, *z* = 1.88, *p* = 0.060, *Nagelkerke R*^*2*^ = 0.09) (Table 2).

**Table 2:** Descriptive distribution (*N*(%) of CM and maternal attachment and test statistic of path 1 (*N* = 103)

	CM +	CM-	test statistic
secure	12 (12)	23 (22)	
insecure	37 (36)	31 (30)	*OR* = 2.46 [0.98, 6.53], *SE* = 0.48, *z* = 1.88, *p* = .060

Model was calculated with the inclusion of the control variables mother's age, child's age, child's sex, mother's years of education, and mothers stress level

**Table 3:** Multiple regression model of path 2 (*N* = 103)

	*β*	*SE*	*t*	*p*	
mother–child-interaction					
CM	0.33	0.20	1.65	.103	
maternal attachment	1.75	0.20	8.73	< .001***	*F*(8, 94) = 11.46, *p* < .001***, *R*^*2*^ = 45 %

Model was calculated with the inclusion of the control variables mother's age, child's age, child's sex, mother's years of education, and mothers stress level; *** *p* < .001

**Table 4:** Multiple regression model of path 3 (*N* = 103)

	*β*	*SE*	*t*	*p*	
3.1 helplessness					
CM	1.21	0.74	1.62	.108	
maternal attachment	0.23	1.00	0.23	.820	
mother–child-interaction	1.29	0.39	3.33	.001**	*F*(9, 93) = 8.62, *p* < .001***, *R*^*2*^ = 40 %
3.2 fear					
CM	0.42	0.62	0.68	.498	
maternal attachment	0.08	0.67	0.13	.900	
mother–child-interaction	1.53	0.28	5.44	< .001***	*F*(9, 93) = 7.40, *p* < .001***, *R*^*2*^ = 36 %
3.3 role reversal					
CM	0.19	0.63	0.30	.765	
maternal attachment	-0.35	0.84	-0.42	.675	
mother–child-interaction	-1.07	0.32	-3.36	.001**	*F*(9, 93) = 3.75, *p* < .001***, *R*^*2*^ = 20 %

Model was calculated with the inclusion of the control variables mother's age, child's age, child's sex, mother's years of education, and mothers stress level; ** *p* < .01, *** *p* < .001

**Table 5:** Multiple regression model of path 4 with maternal helplessness as predictor (*N* = 103)

	*β*	*SE*	*t*	*p*	
4.1.1 emotional problems					
CM	-0.60	0.32	-1.89	.062	
maternal attachment	0.05	0.38	0.14	.889	
mother–child-interaction	0.01	0.17	0.03	.974	
helplessness	0.09	0.04	1.97	.052	*F*(10, 92) = 2.87, *p* = .004**, *R*^*2*^ = 15 %
4.1.2 conduct problems					
CM	-0.20	0.30	-0.66	.514	
maternal attachment	0.20	0.39	0.51	.609	
mother–child-interaction	-0.08	0.16	-0.50	.618	
helplessness	0.11	0.04	2.62	.010*	*F*(10, 92) = 3.57, *p* = .001**, *R*^*2*^ = 20 %
4.1.3 hyperactivity					
CM	0.19	0.44	0.44	.661	
maternal attachment	0.54	0.58	0.92	.356	
mother–child-interaction	0.21	0.24	0.87	.387	
helplessness	0.05	0.06	0.87	.386	*F*(10, 92) = 1.78, *p* = .075, *R*^*2*^ = 7 %
4.1.4 peer problems					
CM	-0.03	0.34	-0.09	.932	
maternal attachment	-0.13	0.45	-0.29	.773	
mother–child-interaction	0.08	0.18	0.44	.664	
helplessness	0.08	0.05	1.73	.087	*F*(10, 92) = 0.86, *p* = .574, *R*^*2*^ = 0 %
4.1.5 prosocial behavior					
CM	0.51	0.39	1.31	.194	
maternal attachment	-0.14	0.52	-0.27	.790	
mother–child-interaction	0.00	0.21	0.01	.994	
helplessness	-0.02	0.06	-0.42	.672	*F*(10, 92) = 1.94, *p* = .049*, *R*^*2*^ = 8 %
4.1.6 overall scale					
CM	0.05	0.74	0.07	.941	
maternal attachment	0.51	0.97	0.53	.601	
mother–child-interaction	0.33	0.39	0.84	.405	
helplessness	0.29	0.10	2.75	.001**	*F*(10, 92) = 3.98, *p* < .001***, *R*^*2*^ = 23 %

Model was calculated with the inclusion of the control variables mother's age, child's age, child's sex, mother's years of education, and mothers stress level; * *p* < .05; ** *p* < .01. *** *p* < .001

**Table 6:** Multiple regression model of path 4 with maternal and child fear as predictor (*N* = 103)

	*β*	*SE*	*t*	*p*	
4.2.1 emotional problems					
CM	-0.55	0.30	-1.83	.070	
maternal attachment	0.06	0.38	0.16	.874	
mother–child-interaction	-0.10	0.17	-0,58	.562	
fear	0.14	0.05	2.81	.006**	*F*(10, 92) = 3.54, *p* < .001***, *R*^*2*^ = 19 %
4.2.2 conduct problems					
CM	-0.09	0.30	-0.29	.773	
maternal attachment	0.22	0.40	0.55	.585	
mother–child-interaction	-0.03	0.17	-0.18	.860	
fear	0.06	0.05	1.16	.247	*F*(10, 92) = 2.86, *p* = .003**, *R*^*2*^ = 15 %
4.2.3 hyperactivity					
CM	0.24	0.44	0.54	.591	
maternal attachment	0.55	0.58	0.94	.350	
mother–child-interaction	0.19	0.25	0.75	.458	
fear	0.06	0.08	0.78	.440	*F*(10, 92) = 1.76, *p* = .078, *R*^*2*^ = 7 %
4.2.4 peer problems					
CM	.04	0.34	0.11	.911	
maternal attachment	-0.12	0.45	-0.26	.795	
mother–child-interaction	0.07	0.20	0.34	.734	
fear	0.08	0.06	1.35	.180	*F*(10, 92) = 0.74, *p* = .689, *R*^*2*^ = 0 %
4.2.5 prosocial behavior					
CM	0.49	0.39	1.25	.213	
maternal attachment	-0.14	0.52	-0.28	.783	
mother–child-interaction	-0.02	0.22	-0.09	.928	
fear	-0.01	0.07	-0.08	.935	*F*(10, 92) = 1.92, *p* = .052, *R*^*2*^ = 8 %
4.2.6 overall scale					
CM	0.26	0.73	0.36	.720	
maternal attachment	0.55	0.98	0.56	.576	
mother–child-interaction	0.20	0.42	0.48	.634	
fear	0.33	0.13	2.59	.011*	*F*(10, 92) = 3.87, *p* < .001***, *R*^*2*^ = 22 %

Model was calculated with the inclusion of the control variables mother's age, child's age, child's sex, mother's years of education, and mothers stress level; * *p* < .05; ** *p* < .01, *** *p* < .001

**Table 7:** Multiple regression model of path 4 with child role reversal as predictor (*N* = 103)

	*β*	*SE*	*t*	*p*	
4.3.1 emotional problems					
CM	-0.48	0.30	-1.59	.116	
maternal attachment	0.06	0.41	0.14	.889	
mother–child-interaction	0.07	0.16	0.43	.666	
role reversal	-0.04	0.05	-0.88	.380	*F*(10, 92) = 2.44, *p* = .013**, *R*^*2*^ = 12 %
4.3.2 conduct problems					
CM	-0.05	0.30	-0.16	.870	
maternal attachment	0.20	0.40	0.50	.616	
mother–child-interaction	-0.01	0.16	-0.06	.951	
role reversal	-0.07	0.05	-1.34	.183	*F*(10, 92) = 2.92, *p* = .003**, *R*^*2*^ = 16 %
4.3.3 hyperactivity					
CM	0.25	0.44	0.58	.565	
maternal attachment	0.57	0.59	0.97	.334	
mother–child-interaction	0.32	0.24	1.36	.176	
role reversal	0.04	0.07	0.60	.550	*F*(10, 92) = 1.74, *p* = .084, *R*^*2*^ = 7 %
4.3.4 peer problems					
CM	0.10	0.33	0.30	.774	
maternal attachment	-0.16	0.45	-0.35	.725	
mother–child-interaction	0.05	0.18	0.26	.794	
role reversal	-0.13	0.06	-2.37	.020*	*F*(10, 92) = 1.14, *p* = .344, *R*^*2*^ = 1 %
4.3.5 prosocial behavior					
CM	0.46	0.38	1.21	.231	
maternal attachment	-0.09	0.48	-0.19	.854	
mother–child-interaction	0.14	0.19	0.74	.458	
role reversal	0.16	0.06	2.57	.012*	*F*(10, 92) = 2.69, *p* = .006**, *R*^*2*^ = 14 %
4.3.6 overall scale					
CM	0.41	0.76	0.54	.592	
maternal attachment	0.56	1.01	0.56	.578	
mother–child-interaction	0.66	0.41	1.63	.108	
role reversal	-0.03	0.13	-0.25	.800	*F*(10, 92) = 2.99, *p* = .003**, *R*^*2*^ = 46 %

Model was calculated with the inclusion of the control variables mother's age, child's age, child's sex, mother's years of education, and mothers stress level; * *p* < .05; ** *p* < .01

In the second path of the path model, maternal attachment representation was a significant predictor for mother–child-interaction. Mothers with insecure attachment representation had higher *AMBIANCE*-scores than mothers with secure attachment representation (*β* = 1.74, *SE* = 0.20 *t* = 8.58, *p* =  < .001). The overall model fit was significant (*F*(8, 94) = 11.46, *p* < .001) and explained 45 % of variance.

In the third path of the path model, mother–child-interaction was a significant predictor for helplessness, fear and role reversal. Higher *AMBIANCE* scores predicted higher levels of helplessness (*β* = 1.29, *SE* = 0.39 *t* = 3.33, *p* = < .001) and fear (*β* = 1.53, *SE* = 0.28 *t* = 5.44, *p* = < .001) and lower levels of role reversal (*β* = -1.07, *SE* = 0.32, *t* = -3.36, *p* = < .001). The overall model fit in all three models was significant (*F*(9, 93) = 8.62, *p* < .001; *F*(9, 93) = 7.40, *p* <.001; *F*(9, 93) = 3.75, *p* < .001) with an explanation of the variance of 40 %, 36 % and 20 % (Table 4).

In the fourth path of the path model, maternal helplessness was a significant predictor for conduct problems in children and the overall *SDQ* score. Higher helplessness scores predicted higher levels of conduct problems (*β* = 0.11, *SE* = 0.04, *t* = 2.62, *p* = .010) and a higher overall *SDQ* scale (*β* = 0.29, *SE* = 0.10, *t* = 2.75, *p* = < .001). The overall model fit in both models was significant (*F*(10, 92) = 3.57, *p* < .001; *F*(10, 92) = 3.98, *p* < .001) with an explanation of the variance of 20 % and 23 %. The predictor helplessness did not reach significance in all other calculated models (Table 5). Fear in mother and child was a significant predictor for emotional problems in children and the overall *SDQ* score. Higher levels of fear predicted higher levels of emotional problems (*β* = 0.14, *SE* = 0.05, *t* = 2.81, *p* = .006) and a higher overall *SDQ* scale (*β* = 0.33, *SE* = 0.13, *t* = 2.59, *p* = 0.011). The overall model fit in both models was significant (*F*(10, 92) = 3.54, *p* < .001; *F*(10, 92) = 3.87, *p* < .001) with an explanation of the variance of 19 % and 22 %. The predictor fear did not reach significance in all other calculated models (Table 6). Role reversal in children was a significant predictor for prosocial behavior in children. Higher levels of role reversal predicted higher levels of prosocial behavior (*β* = 0.16, *SE* = 0.06, *t* = 2.57, *p* = .012). The overall model fit was significant (*F*(10, 92) = 2.69, *p* = .006). Additionally, role reversal became a significant negative predictor for peer problems (*β* = -0.13, *SE* = 0.06, *t* = -2.37, *p* = .020). However the model fit was not significant (*F*(10, 92) = 1.14, *p* = .344, *adjusted R*^*2*^ = 0.01). The predictor role reversal did not reach significance in all other calculated models (Table 7).

## Discussion

This study investigated the pathways between maternal CM experiences, maternal attachment representation, mother–child-interaction one year after birth, maternal helplessness, fear and children’s mental health in kindergarten age. We were able to uncover a link between maternal attachment representation and mother–child interaction. Mother–child interaction, in turn, had an impact on mother helplessness, fear in mother and child and child role reversal. Maternal helplessness was positively associated with child conduct problems and a global score of psychological distress. Fear in mother and child was positively associated with emotional problems and a global score of psychological distress. Child role reversal was positively associated with prosocial behavior. No significant relationship could be found between maternal CM experience and maternal attachment representation. Therefore, we were only able to confirm parts of the exploratory path model, as shown in Fig. 2. The individual paths of the model are discussed in detail below.

In this study, maternal CM experiences had no significant effect on maternal attachment representation, in contrast to previous studies [12–14, 45]. However, unlike previous studies [12–14], our study only distinguished between secure and insecure attachment representations. Therefore, this study did not consider the marginalized group of mothers with fearful-avoidant attachment, which can be considered a risk group in isolation. Additionally, previous studies distinguished between different types of abuse, e.g. childhood neglect and physical abuse [12, 14], whereas in our study all types were combined. For instance, Widom and colleagues [12] found that childhood neglect was associated with an anxious attachment style, whereas physical abuse in childhood was associated with an avoidant attachment style. Therefore, a more differentiated consideration of the variables could possibly have led to a significant association between individual factors of the variables.

We were able to demonstrate that attachment representation had a significant impact on mother–child interaction. Mothers with an insecure attachment representation had higher scores on the *AMBIANCE* global score, indicative of more frequent and more intense disrupted behaviors during mother–child-interaction. This finding is consistent with the results of previous studies that showed associations of insecure parental attachment with less sensitivity and intrusive or even harmful parenting behavior, especially under increased stress [15, 17, 46, 47]. In our analyses, maternal stress was included as a covariate, which is why the behavior cannot be attributed to increased stress levels in mothers. It is assumed that mothers with an insecure attachment representation have difficulties to perceive the needs of their children and to react adequately to them. They generally react less sensitively, for example, they react punitively and attribute emotional reactions such as crying to negative characteristics of the children [48, 49]. However, it must be emphasized that with the classification between secure and insecure attachment representation alone, an association with mother–child-interaction behavior could be found. Therefore, it is obvious that even larger effects could be expected in risk samples with unresolved attached mothers.

In the next step of the path analysis, an association of mother–child interaction with maternal helplessness, mother and child anxiety and child role reversal was found. Mothers who showed more disrupted behavior when the child was approximately one year old indicated more helplessness and fear when the child was kindergarten age. It can be assumed that self-efficacy plays a crucial role in the relationships shown in this study. Mothers with insecure attachment style are not able to establish adequate interaction behavior with their child. It is suspected that this is due to their own childhood experiences and the difficulties in establishing new attachments [48, 49]. The effects of a disrupted interaction on the child may be perceived by the mother and therefore lead to a negative impact on her self-efficacy. As noted in Kohlhoff and colleagues' [24] study, CM experiences and insecure attachment representation are risk factors for low self-efficacy. It can be assumed that this low self-efficacy can increase the feeling of helplessness and fear of mothers within their caregiving role. Additionally, the time span between the survey of interactional behavior and the self-assessment of helplessness and fear was several years, suggesting that a maladaptive parenting style may stabilize over years if it is established in the early years of parenting. Taking a closer look at the items of the *CHQ* [18], it can also be assumed that the interaction behavior and its perception of the mother, which leads to helplessness and fear, influence each other and thus cause a vicious circle. For instance, as it is queried in the *CHQ* [18], the mother may view the child as completely out of control and may therefore assume that any care provided does not seem to matter leading to more maladaptive parenting [20]. Some children, who experience disruptive parenting from a young age also tend to show controlling behavior (e.g. role-reversal) as a way to keep the mother engaged in the relationship [50], which would also explain the association of disrupted interaction in this study with the scale role reversal.

In the last step of our path analysis we investigated the impact of maternal helplessness and fear on children's mental health. The results imply that maternal helplessness and fear predict psychological problems in children. More specifically, helplessness was a significant predictor for conduct problems and fear was a significant predictor for emotional problems in both mother and child. As mentioned above, constant feelings of helplessness and fear as a parent can lead to giving up in the parental role failing to protect her child [18], which has a negative impact on children's mental health [25]. Helplessness in mothers showed a significant relation with the *SDQ*-scale conduct problems. This association might be explained by parenting behavior. It can be assumed that helpless mothers, who have already resigned in their efforts of parenting tend to show little consistent parenting behavior with little parental control. This parenting style, especially in combination with low maternal sensitivity represents a risk factor for the development of behavioral disorders, such as conduct disorders and ADHD [51]. The association of fear in mothers with particularly emotional problems is consistent with previous findings showing that fear in mothers can predict anxiety and depressive symptoms in children [52]. Additionally, role reversal was shown to be a significant predictor of child’s prosocial behavior. When looking more closely at the respective items of the *CHQ* and *SDQ* scales, this result is not surprising, as the items show clear overlaps (e.g. *“My child is good at tending to and caring for others”* and “*Often offers to help others (parents, teachers, other children)”*). However, no particular focus was placed on the consideration of the role reversal scale in this study, which is why the result will not be discussed further.

### Limitations and strengths of the study

Considering the present study, several limitations need to be addressed. First, it is important to note that a great number of our participants had a high standard of education, which has not been reported in other German cohort studies [53, 54]. Therefore, the present cohort does not represent the general population, indicating that generalizability of the results might be limited. Second, parts of the data collected is based on self-report questionnaires, which were filled out independently by the mothers. Additionally, mental problems of the children were filled out by the mothers as an external assessment. Therefore, it must be considered that the mothers completed the questionnaires according to social desirability, which may have biased the results of this study. Third, the *AAP* was only evaluated by one coder. Therefore, the reliability of the results from the AAP could be limited. Fourth, unlike in previous studies, the variables CM [12, 14] and maternal attachment [12–14] were each distinguished by only two forms. A more differentiated consideration of CM (e.g. distinction between emotional and physical abuse) and maternal attachment (e.g. distinction between secure, insecure, and unresolved) could have led to more specific results, thereby deriving more concrete implications. Fifth, other variables might play a role in the calculated pathway that have not been included and therefore have confounded the results. For example, the role of the father has not been considered in the present model, unlike previous studies [16]. Of particular interest would be how the consequences of insecure attachment representation and disrupted parenting behaviors of mothers can be countered by adequate parenting and the attachment representation of the father. Sixth, it must be noted that the SDQ is primarily a screening instrument but not a diagnostic instrument for psychiatric disorders. The individual scales do not have a sufficiently high internal consistency to allow reliable statements to be made about individual mental disorders. Children and adolescents are more often wrongly classified as inconspicuous although mental disorders are present [55, 56]. As a final point, the *CHQ* and the *SDQ* were both collected at measuring point t_4_. Therefore, no conclusion can be drawn as to which variable may have influenced which. In addition, the entire study is based on an exploratory approach with an insufficiently large sample. No experimental manipulation of the conditions took place in this study, which implies that causality cannot be assumed. Future studies should consider the points noted. Despite the many limitations, strengths of the study should also be emphasized. The sample in this study was followed over several years and several measurement points. The longitudinal approach of the study can therefore be seen as a strength.

## Conclusions

This study is based on data from a longitudinal cohort study with recruitment of mothers and children at the time of birth and several subsequent measurement time points. The results of our study imply that insecure attachment representation in mothers is associated with disrupted parenting behavior at the age of approximately one year, which in turn predicts helplessness and fear in parenting at preschool age. Additionally, helplessness and fear in mothers were associated with mental health problems, mainly emotional problems and conduct problems. The results of this study highlight how insecure attachment in mothers has a long-term impact on their parenting behaviors and the mental health of their children. It is important to emphasize that the subsample of mothers with insecure attachment representation does not represent a risk sample. Even higher negative effects on maternal behavior could be assumed in a risk sample of mothers with unresolved attachment. The results of this study underscore the importance of early intervention programs, such as EPB (Entwicklungspsychologische Beratung; [57]) and STEEP (Steps Toward Effective and Enjoyable Parenting; [58]) that focus on early mother-infant interactions. However, future studies should investigate to what extent the results of this study can be replicated in a risk sample with a sufficient sample size and can be influenced by additional factors, mainly what the influence father may have in this setting.

## Data Availability

The datasets used and analyzed during the current study are available from the corresponding author on reasonable request.

## References

[1] Gardner MJ, Thomas HJ, Erskine HE (2019). The association between five forms of child maltreatment and depressive and anxiety disorders: A systematic review and meta-analysis. Child Abuse Negl.

[2] Humphreys KL, LeMoult J, Wear JG, Piersiak HA, Lee A, Gotlib IH (2020). Child maltreatment and depression: A meta-analysis of studies using the Childhood Trauma Questionnaire. Child Abuse Negl.

[3] Malta LA, McDonald SW, Hegadoren KM, Weller CA, Tough SC (2012). Influence of interpersonal violence on maternal anxiety, depression, stress and parenting morale in the early postpartum: a community based pregnancy cohort study. BMC Pregnancy Childbirth.

[4] Madigan S, Cyr C, Eirich R, Fearon RP, Ly A, Rash C., ... & Alink LR. Testing the cycle of maltreatment hypothesis: Meta-analytic evidence of the intergenerational transmission of child maltreatment. Dev Psychopathol 2019;31(1):23–51. 10.1017/S0954579418001700. 10.1017/S0954579418001700 30757994

[5] Savage LÉ, Tarabulsy GM, Pearson J, Collin-Vézina D, Gagné LM (2019). Maternal history of childhood maltreatment and later parenting behavior: A meta-analysis. Dev Psychopathol.

[6] Walden ED, Hamilton JC, Harrington E, Lopez S, Onofrietti-Magrassi A, Mauricci M, Trevino S, Giuliani N, McIntyre LL (2022). Intergenerational Trauma: Assessment in Biological Mothers and Preschool Children. J Child Adolesc Trauma.

[7] Ehrensaft MK, Knous-Westfall HM, Cohen P, Chen H (2015). How does child abuse history influence parenting of the next generation?. Psychol Violence.

[8] Köhler-Dauner F, Clemens V, Hildebrand K, Ziegenhain U, Fegert JM (2021). The interplay between maternal childhood maltreatment, parental coping strategies as well as endangered parenting behavior during the current SARS-CoV-2 pandemic. Developmental child welfare.

[9] Grand, S. & Salberg, J. Trans-Generational Transmission of Trauma. In A. Hamburger, C. Hancheva & V. D. Volkan (Hrsg.), *Social Trauma – An Interdisciplinary Textbook* (S. 209–215). (2021). Springer International Publishing. 10.1007/978-3-030-47817-9_22.

[10] Özcan NK, Boyacioğlu NE, Enginkaya S, Bilgin H, Tomruk NB (2016). The relationship between attachment styles and childhood trauma: a transgenerational perspective - a controlled study of patients with psychiatric disorders. J Clin Nurs.

[11] Köhler-Dauner F, Buchheim A, Hildebrand K, Mayer I, Clemens V, Ziegenhain U, Fegert JM (2022). Maternal attachment representation, the risk of increased depressive symptoms and the influence on children's mental health during the SARS-CoV-2-pandemic. J Child Fam Stud.

[12] Widom CS, Czaja SJ, Kozakowski SS, Chauhan P (2018). Does adult attachment style mediate the relationship between childhood maltreatment and mental and physical health outcomes?. Child Abuse Negl.

[13] Shahab MK, de Ridder JA, Spinhoven P, Penninx BW, Mook-Kanamori DO, Elzinga BM (2021). A tangled start: The link between childhood maltreatment, psychopathology, and relationships in adulthood. Child Abuse Negl.

[14] Unger JAM, De Luca RV (2014). The Relationship Between Childhood Physical Abuse and Adult Attachment Styles. Journal of Family Violence.

[15] Mills-Koonce WR, Appleyard K, Barnett M, Deng M, Putallaz M, Cox M (2011). Adult Attachment Style and Stress as Risk Factors for Early Maternal Sensitivity and Negativity. Infant Ment Health J.

[16] Zvara BJ, Lathren C, Mills-Koonce R (2020). Maternal and paternal attachment style and chaos as risk factors for parenting behavior. Fam Relat.

[17] Moe V, von Soest T, Fredriksen E, Olafsen KS, Smith L (2018). The Multiple Determinants of Maternal Parenting Stress 12 Months After Birth: The Contribution of Antenatal Attachment Style, Adverse Childhood Experiences, and Infant Temperament. Front Psychol.

[18] George C, Solomon J, Solomon J, George C (2011). Caregiving helplessness: The development of a screening measure for disorganized maternal caregiving. Disorganized attachment and caregiving.

[19] Bernstein R. Identifying Perinatal Predictors of Disorganized Infant-Mother Attachment: An Important Step Toward Connecting Families with Appropriate Early Interventions, University of Oregon. (2016).

[20] Huth-Bocks AC, Guyon-Harris K, Calvert M, Scott S, Ahlfs-Dunn S (2016). The caregiving helplessness questionnaire: evidence for validity and utility with mothers and infants. Infant Ment Health J.

[21] Draxler H, Hjärthag F, Almqvist K (2019). Replicability of effect when transferring a supportive programme for parents exposed to intimate partner violence and their children from the US to Sweden. Child Care Pract.

[22] Grip K. Intimate Partner Violence Victimization, Maternal harsh discipline, and the Mediating Impact of care-giving helplessness and parental control. Arch Psychol. 2019;3(1).

[23] Caldwell JG, Shaver PR, Li C-S, Minzenberg MJ (2011). Childhood maltreatment, adult attachment, and depression as predictors of parental self-efficacy in at-risk mothers. J Aggression Maltreatment Trauma.

[24] Kohlhoff J, Barnett B (2013). Parenting self-efficacy: links with maternal depression, infant behaviour and adult attachment. Early Human Dev.

[25] Möller EL, Majdandžić M, Bögels SM (2015). Parental Anxiety, Parenting Behavior, and Infant Anxiety: Differential Associations for Fathers and Mothers. J Child Fam Stud.

[26] Huizink AC, Menting B, De Moor MHM, Verhage ML, Kunseler FC, Schuengel C, Oosterman M (2017). From prenatal anxiety to parenting stress: a longitudinal study. Arch Womens Ment Health.

[27] Köhler-Dauner F, Clemens V, Lange S, Ziegenhain U, Fegert JM (2021). Mothers' daily perceived stress influences their children's mental health during SARS-CoV-2-pandemic-an online survey. Child Adolesc Psychiatry Ment Health.

[28] de Maat DA, Jansen PW, Prinzie P, Keizer R, Franken IHA, Lucassen N (2021). Examining longitudinal relations between mothers’ and fathers’ parenting stress, parenting behaviors, and adolescents’ behavior problems. J Child Fam Stud.

[29] Mackler JS, Kelleher RT, Shanahan L, Calkins SD, Keane SP, O'Brien M (2015). Parenting stress, parental reactions, and externalizing behavior from ages 4 to 10. J Marriage Fam.

[30] Bader K, Hänny C, Schäfer V, Neuckel A, Kuhl C (2009). Childhood Trauma Questionnaire – Psychometrische Eigenschaften einer deutschsprachigen Version. Z Klin Psychol Psychother.

[31] George C, West ML (2012). The Adult Attachment Projective Picture System: Attachment theory and assessment in adults.

[32] Bronfman, E., Madigan, S [S.] & Lyons-Ruth, K. (2009). Disrupted Maternal Behavior Instrument for Assessment and Classification (AMBIANCE): Manual for coding disrupted affective communication. Unpublished manuscript, Harvard University Medical School.

[33] Lyons-Ruth, K., Bureau, J.F., Holmes, B., Easterbrooks, A., & Brooks, N.H. (2013). Borderline symptoms and suicidality/self-injury in late adolescence: Prospectively observed relationship correlates in in fancy and childhood. Psychiatry Research, 206(2–3), 273–281. Klasen, H., Woerner, W., Rothenberger, A. & Goodman, R. (2003). Die deutsche Fassung des Strengths and Difficulties Questionnaire (SDQ-Deu) - Übersicht und Bewertung erster Validierungs- und Normierungsbefunde*.*10.23668/PSYCHARCHIVES.11726.

[34] Klinitzke G, Romppel M, Häuser W, Brähler E, Glaesmer H (2012). Die deutsche Version des Childhood Trauma Questionnaire (CTQ) - psychometrische Eigenschaften in einer bevölkerungsrepräsentativen Stichprobe [The German Version of the Childhood Trauma Questionnaire (CTQ): psychometric characteristics in a representative sample of the general population]. Psychother Psychosom Med Psychol.

[35] George, C., Kaplan, N. & Main, M [M.]. (1996). Adult Attachment Interview.

[36] Beliveau MJ, Moss E. Validation du projectif de l'attachement adulte (AAP): Contribution aux validités convergente et divergente du projectif de l'attachement adulte. *La Revue internationale de l'éducation familiale.* 2005;9(1).

[37] Bronfman E, Parsons E, Lyons-Ruth K. Atypical Maternal Behavior Instrument for Assessment and Classification (AMBIANCE): Manual for coding affective communication. Unpublished manual. Cambridge, MA: Department of Psychiatry, Harvard Medical School; 1992–2009.

[38] Main, M. & Hesse, E. (1990). Parents' unresolved traumatic experiences are related to infant disorganized attachment status: Is frightened and/or frightening parental behavior the linking mechanism? In The John D. and Catherine T. MacArthur Foundation series on mental health and development. Attachment in the preschool years: Theory, research, and intervention (S. 161–182). The University of Chicago Press.

[39] Husky MM, Otten R, Boyd A, Pez O, Bitfoi A, Carta MG, Goelitz D, Koç C, Lesinskiene S, Mihova Z, Kovess-Masfety V (2020). Psychometric properties of the strengths and difficulties questionnaire in children aged 5–12 years across seven European Countries. Eur J Psychol Assess.

[40] Cohen, S., Kamarck, T. & Mermelstein, R. A Global Measure of Perceived Stress.J Health Soc Behav. 1983;24(4):385–396. 10.2307/2136404 —> Im Text stand 1995. 6668417

[41] Andreou E, Alexopoulos EC, Lionis C, Varvogli L, Gnardellis C, Chrousos GP, Darviri C (2011). Perceived stress scale: reliability and validity study in greece. Int J Environ Res Public Health.

[42] Katsarou, A., Panagiotakos, D., Zafeiropoulou, A., Vryonis, M., Skoularigis, I., Tryposkiadis, F. & Papageorgiou, C. Validation of a Greek version of PSS-14; a global measure of perceived stress. Central Eur J Public Health. 2012;20(2):104–109. 10.21101/cejph.a3698. 10.21101/cejph.a3698 22966732

[43] R Core Team. (2022). R: A language and environment for statistical computing. R Foundation for Statistical Computing, Vienna, Austria.

[44] Hayes AF, Cai L (2007). Using heteroskedasticity-consistent standard error estimators in OLS regression: an introduction and software implementation. Behav Res Methods.

[45] Buchheim A, Ziegenhain U, Kindler H, Waller C, Gündel H, Karabatsiakis A, Fegert J (2022). Identifying risk and resilience factors in the intergenerational cycle of maltreatment: results from the TRANS-GEN study investigating the effects of maternal attachment and social support on child attachment and cardiovascular stress physiology. Front Hum Neurosci.

[46] Gulde M, Köhler-Dauner F, Mayer I, Ziegenhain U, Fegert JM, Buchheim A (2022). Negative effects of the SARS-CoV-2 pandemic: The interlinking of maternal attachment representation, coping strategies, parental behavior, and the child's mental health. Front Pediatr.

[47] Zvara, B. J., Lathren, C., Mills‐Koonce, R., & Family Life Project Key Contributors. (2020). Maternal and paternal attachment style and chaos as risk factors for parenting behavior. Family relations. 2020;69(2):233-246 10.1111/fare.12423 PMC8061620 33897080

[48] Gross, J. T., Stern, J. A., Brett, B. E., Fitter, M. H. & Cassidy, J. Mothers’ Attachment Style Predicts Response to Child Distress: The Role of Maternal Emotions and Attributions. *Journal of Child and Family Studies.* Vorab-Onlinepublikation. 2022 . 10.1007/s10826-022-02517-5. 10.1007/s10826-022-02517-5 PMC10586594 37859978

[49] Pickard JA, Townsend M, Caputi P, Grenyer BFS (2017). Observing the influence of mindfulness and attachment styles through mother and infant interaction: a longitudinal study. Infant Ment Health J.

[50] Solomon J, George C, Solomon J, George C (2011). The disorganized attachment-caregiving system: Dysregulation of adaptive processes at multiple levels. Disorganized attachment and caregiving.

[51] Popow, C. & Ohmann, S. ADHS im Kindes- und Jugendalter. Update 2020. Pädiatrie Pädologie. 2020;55(S1);1–22. 10.1007/s00608-020-00789-y

[52] Telman LGE, van Steensel FJA, Maric M, Bögels SM (2018). What are the odds of anxiety disorders running in families? A family study of anxiety disorders in mothers, fathers, and siblings of children with anxiety disorders. Eur Child Adolesc Psychiatry.

[53] Kantorczyk E, Domanski G, Lange AE, Ittermann T, Allenberg H, Zygmunt M, Heckmann M (2020). Survey of Neonates in Pomerania (SNiP): Study design and cohort update. Paediatr Perinat Epidemiol.

[54] Kohlhuber M, Rebhan B, Schwegler U, Koletzko B, Fromme H (2008). Breastfeeding rates and duration in Germany: a Bavarian cohort study. Br J Nutr.

[55] Bettge S, Ravens-Sieberer U, Wietzker, A. & Hölling, H. Ein Methodenvergleich der Child Behavior Checklist und des Strengths and Difficulties Questionnaire. Das Gesundheitswesen. 2002;64:119-24. 10.1055/s-2002-39264. 10.1055/s-2002-39264 12870226

[56] Brøndbo PH, Mathiassen B, Martinussen M, Heiervang E, Eriksen M, Moe TF, Sæther G, Kvernmo S (2011). The strengths and difficulties questionnaire as a screening instrument for norwegian child and adolescent mental health services, application of UK scoring algorithms. Child Adolesc Psychiatry Ment Health.

[57] Ziegenhain, U., Fries, M., Bütow, B., & Derksen, B. Entwicklungspsychologische Beratung für junge Eltern: Grundlagen und Handlungskonzepte für die Jugendhilfe. Familienbildung und Beratung. Juventa. 2004. http://www.socialnet.de/rezensionen/isbn.php?isbn=978-3-7799-1533-1.

[58] Suess, G. J., Erickson, M. F., Egeland, B., Scheuerer‑Englisch, H. & Hartmann, H.‑P. (2018). Steps toward effective, enjoyable parenting: Lessons from 30 years of implementation, adaptation, and evaluation. In Handbook of attachment-based interventions (S. 104–128). The Guilford Press.

